# Supplementary material for: Modeling glioblastoma heterogeneity as a dynamic network of cell states
Source: Mol Syst Biol. 2021 Sep 16;17(9):e10105. doi: 10.15252/msb.202010105 (PMC8444284; doi:10.15252/msb.202010105)
Supplement: Supplementary file 6 — Source Data for Figure 5 [file MSB-17-e10105-s004.zip › Figure5A_sourcedata/GSEA_3017/nosigdb_stateA.GseaPreranked.1622795026485/RICHARDS_ZHONG_NATURE_2018_ASTROCYTES_UPREG.html]

Details for gene set RICHARDS\_ZHONG\_NATURE\_2018\_ASTROCYTES\_UPREG[GSEA]

|  || Dataset | state53017 |
| Phenotype | NoPhenotypeAvailable |
| Upregulated in class | na\_neg |
| GeneSet | RICHARDS\_ZHONG\_NATURE\_2018\_ASTROCYTES\_UPREG |
| Enrichment Score (ES) | -0.44244963 |
| Normalized Enrichment Score (NES) | -2.6670003 |
| Nominal p-value | 0.0 |
| FDR q-value | 0.0 |
| FWER p-Value | 0.0 |
Table: GSEA Results Summary

  

Fig 1: Enrichment plot: RICHARDS\_ZHONG\_NATURE\_2018\_ASTROCYTES\_UPREG      
 Profile of the Running ES Score & Positions of GeneSet Members on the Rank Ordered List

  

| PROBE | GENE SYMBOL | GENE\_TITLE | RANK IN GENE LIST | RANK METRIC SCORE | RUNNING ES | CORE ENRICHMENT || 1 | FABP5 |  |  | 0 | 1.759 | 0.0408 | No |
| 2 | DBI |  |  | 17 | 0.683 | 0.0390 | No |
| 3 | CA12 |  |  | 24 | 0.645 | 0.0473 | No |
| 4 | VIM |  |  | 46 | 0.543 | 0.0366 | No |
| 5 | S100A16 |  |  | 156 | 0.404 | -0.0748 | No |
| 6 | TNC |  |  | 159 | 0.402 | -0.0677 | No |
| 7 | SERPINE2 |  |  | 201 | 0.373 | -0.1045 | No |
| 8 | ALCAM |  |  | 252 | 0.348 | -0.1519 | No |
| 9 | BCAN |  |  | 276 | 0.334 | -0.1696 | No |
| 10 | HSPB1 |  |  | 312 | 0.322 | -0.2009 | No |
| 11 | IDI1 |  |  | 334 | 0.314 | -0.2169 | No |
| 12 | ATP13A4 |  |  | 410 | 0.290 | -0.2933 | No |
| 13 | P4HA1 |  |  | 411 | 0.290 | -0.2866 | No |
| 14 | NT5E |  |  | 445 | 0.282 | -0.3166 | No |
| 15 | BCHE |  |  | 456 | 0.280 | -0.3212 | No |
| 16 | SLITRK2 |  |  | 458 | 0.279 | -0.3158 | No |
| 17 | MT2A |  |  | 473 | 0.275 | -0.3250 | No |
| 18 | PSAT1 |  |  | 491 | 0.271 | -0.3375 | No |
| 19 | ZFP36L1 |  |  | 532 | 0.264 | -0.3758 | No |
| 20 | SOX9 |  |  | 577 | 0.254 | -0.4186 | No |
| 21 | AGT |  |  | 587 | 0.252 | -0.4228 | No |
| 22 | CD82 |  |  | 597 | 0.250 | -0.4269 | No |
| 23 | ITGAV |  |  | 599 | -0.250 | -0.4222 | No |
| 24 | SYPL1 |  |  | 608 | -0.255 | -0.4252 | No |
| 25 | ABAT |  |  | 617 | -0.259 | -0.4280 | No |
| 26 | PLIN3 |  |  | 619 | -0.259 | -0.4231 | No |
| 27 | DAG1 |  |  | 622 | -0.260 | -0.4193 | No |
| 28 | GPR37L1 |  |  | 624 | -0.261 | -0.4143 | No |
| 29 | ARHGEF26 |  |  | 628 | -0.262 | -0.4116 | No |
| 30 | SDC3 |  |  | 637 | -0.264 | -0.4143 | No |
| 31 | GNG12 |  |  | 640 | -0.265 | -0.4104 | No |
| 32 | PEA15 |  |  | 668 | -0.281 | -0.4338 | No |
| 33 | SPRY2 |  |  | 669 | -0.282 | -0.4273 | No |
| 34 | PTTG1IP |  |  | 678 | -0.290 | -0.4294 | No |
| 35 | SEMA6A |  |  | 679 | -0.292 | -0.4226 | No |
| 36 | DDR1 |  |  | 683 | -0.294 | -0.4191 | No |
| 37 | EFEMP2 |  |  | 686 | -0.294 | -0.4145 | No |
| 38 | S100B |  |  | 696 | -0.299 | -0.4175 | No |
| 39 | TSC22D4 |  |  | 716 | -0.312 | -0.4314 | No |
| 40 | ID2 |  |  | 727 | -0.323 | -0.4350 | Yes |
| 41 | CBR1 |  |  | 732 | -0.327 | -0.4318 | Yes |
| 42 | C1orf61 |  |  | 733 | -0.328 | -0.4242 | Yes |
| 43 | ELOVL2 |  |  | 735 | -0.329 | -0.4177 | Yes |
| 44 | HES1 |  |  | 736 | -0.331 | -0.4100 | Yes |
| 45 | KLHDC8A |  |  | 741 | -0.336 | -0.4066 | Yes |
| 46 | PON2 |  |  | 743 | -0.338 | -0.3999 | Yes |
| 47 | HEY1 |  |  | 751 | -0.344 | -0.3996 | Yes |
| 48 | C1orf122 |  |  | 753 | -0.345 | -0.3927 | Yes |
| 49 | CYP51A1 |  |  | 759 | -0.349 | -0.3902 | Yes |
| 50 | WSCD1 |  |  | 761 | -0.351 | -0.3831 | Yes |
| 51 | SLC4A4 |  |  | 763 | -0.355 | -0.3760 | Yes |
| 52 | MLC1 |  |  | 783 | -0.369 | -0.3885 | Yes |
| 53 | MSMO1 |  |  | 803 | -0.386 | -0.4006 | Yes |
| 54 | MRC2 |  |  | 813 | -0.395 | -0.4014 | Yes |
| 55 | HOPX |  |  | 821 | -0.403 | -0.3998 | Yes |
| 56 | GJA1 |  |  | 823 | -0.405 | -0.3915 | Yes |
| 57 | DDAH1 |  |  | 828 | -0.413 | -0.3863 | Yes |
| 58 | ZFP36L2 |  |  | 848 | -0.443 | -0.3971 | Yes |
| 59 | ELN |  |  | 852 | -0.445 | -0.3901 | Yes |
| 60 | SLC1A2 |  |  | 860 | -0.450 | -0.3874 | Yes |
| 61 | SERPINB6 |  |  | 861 | -0.452 | -0.3769 | Yes |
| 62 | DOK5 |  |  | 862 | -0.457 | -0.3663 | Yes |
| 63 | JUN |  |  | 886 | -0.525 | -0.3796 | Yes |
| 64 | F3 |  |  | 888 | -0.526 | -0.3685 | Yes |
| 65 | PDPN |  |  | 890 | -0.529 | -0.3574 | Yes |
| 66 | UBL3 |  |  | 893 | -0.535 | -0.3471 | Yes |
| 67 | GPM6B |  |  | 897 | -0.542 | -0.3379 | Yes |
| 68 | ATP1B2 |  |  | 904 | -0.558 | -0.3316 | Yes |
| 69 | MT3 |  |  | 905 | -0.559 | -0.3186 | Yes |
| 70 | DKK3 |  |  | 912 | -0.579 | -0.3118 | Yes |
| 71 | RAB31 |  |  | 916 | -0.586 | -0.3015 | Yes |
| 72 | GLUL |  |  | 920 | -0.612 | -0.2906 | Yes |
| 73 | ID1 |  |  | 931 | -0.651 | -0.2866 | Yes |
| 74 | TTYH1 |  |  | 932 | -0.652 | -0.2715 | Yes |
| 75 | CST3 |  |  | 933 | -0.680 | -0.2557 | Yes |
| 76 | CLU |  |  | 938 | -0.685 | -0.2442 | Yes |
| 77 | LRRC17 |  |  | 949 | -0.769 | -0.2374 | Yes |
| 78 | IFITM3 |  |  | 954 | -0.830 | -0.2226 | Yes |
| 79 | CNN3 |  |  | 961 | -0.883 | -0.2087 | Yes |
| 80 | LGALS3 |  |  | 970 | -0.989 | -0.1946 | Yes |
| 81 | FABP7 |  |  | 971 | -0.994 | -0.1716 | Yes |
| 82 | SLC1A3 |  |  | 973 | -1.097 | -0.1472 | Yes |
| 83 | LRRN3 |  |  | 977 | -1.233 | -0.1219 | Yes |
| 84 | PMP22 |  |  | 982 | -1.519 | -0.0911 | Yes |
| 85 | SPARCL1 |  |  | 984 | -1.810 | -0.0501 | Yes |
| 86 | GFAP |  |  | 987 | -2.255 | -0.0000 | Yes |
Table: GSEA details [plain text format]

  

Fig 2: RICHARDS\_ZHONG\_NATURE\_2018\_ASTROCYTES\_UPREG: Random ES distribution      
 Gene set null distribution of ES for **RICHARDS\_ZHONG\_NATURE\_2018\_ASTROCYTES\_UPREG**
